# Is unilateral pedicle screw fixation superior than bilateral pedicle screw fixation for lumbar degenerative diseases: a meta-analysis

**DOI:** 10.1186/s13018-018-1004-x

**Published:** 2018-11-22

**Authors:** Pei Lu, Ting Pan, Teng Dai, Gang Chen, Ke-qin Shi

**Affiliations:** Orthopedics Department, The Affiliated Wuxi No.2 People’s Hospital of Nanjing Medical University, No. 68 Zhongshan Road, Wuxi, Jiangsu Province 214000 China

**Keywords:** Lumbar degenerative diseases, Unilateral pedicle screw fixation, Bilateral pedicle screw fixation; fusion rate, Meta-analysis

## Abstract

**Background:**

To investigate whether unilateral pedicle screw fixation is superior than bilateral pedicle screw fixation for lumbar degenerative diseases.

**Methods:**

Electronic databases including PubMed, Embase, and the Cochrane Library up to August 2018 were searched. All the high-quality randomized controlled trials (RCTs) and prospective clinical controlled studies comparing the unilateral pedicle screw fixation and bilateral pedicle screw fixation for lumbar degenerative diseases were enrolled in this study. Fusion rate was the main outcome. Stata 12.0 was used for the meta-analysis.

**Results:**

Twelve RCTs including 808 patients (unilateral pedicle screw fixation = 393, bilateral pedicle screw fixation = 415) were included in our meta-analysis. There was a significant difference between unilateral pedicle screw fixation and bilateral pedicle screw fixation in terms of the fusion rate (risk ratio (RR) = 0.96, 95%CI [0.92, 1.00], *P* = 0.073), visual analog scale (VAS) at final follow-up, Oswestry Disability Index (ODI), Japanese Orthopedic Association scores (JOA), short-form health survey (SF-36), and length of hospital stay. Compared with bilateral pedicle screw fixation, unilateral pedicle screw fixation was associated with a reduction of the total blood loss and operation time (*P* < 0.05). Unilateral pedicle screw fixation was associated with an increase of the cage migration than bilateral pedicle screw fixation (17.1% vs 7.1%, RR = 2.40, 95% CI = 1.17 to 4.93; *P* = 0.017).

**Conclusions:**

Unilateral pedicle screw fixation and bilateral pedicle screw fixation has similar fusion rate when treating for lumbar degenerative diseases. Our meta-analysis suggested that compared with bilateral pedicle screw fixation, unilateral pedicle screw fixation significantly reduced total blood loss and operation time for lumbar degenerative diseases. The use of unilateral pedicle screw for lumbar degenerative diseases increases the cage migration.

## Introduction

Lumbar spinal fusion is recognized as an effective surgical procedure for degenerative lumbar diseases [1]. Lumbar fusion can achieve solid arthrodesis, immobilizing the unstable segment and degenerated intervertebral disk area [2]. Bilateral pedicle screw fixation after interbody fusion is regarded as a standard surgical method for degenerative lumbar diseases.

However, rigid fixation also has corresponding shortcomings. Rigid internal fixation may accelerate the degeneration of adjacent lumbar segments and cause device-related osteoporosis. Moreover, bilateral pedicle screw fixation was associated with greater blood loss, longer operative time, and involving greater costs [3].

In 1991, Goel et al. [4] revealed that unilateral pedicle screw fixation could reduce the effects of stress shielding on the fixed vertebrae and avoid adjacent intervertebral disc degeneration. What is more, some scholars reported that unilateral pedicle screw fixation is sufficient to maintain the stability of the spine. A previous biomechanical study revealed that the initial stability of unilateral pedicle screw fixation may be inadequate to obtain improved surgical outcomes.

However, a few controversies remain on whether this method could be applied in lumbar degenerative diseases for the lack of long-term results, such as the fusion rate, perioperative complications, and long-term functional outcomes [5]. Previous meta-analyses articles comparing unilateral and bilateral pedicle screw fixation for lumbar degenerative diseases revealed that unilateral pedicle screw fixation has similar clinical outcomes with bilateral pedicle screw fixation [6]. However, mixed randomized controlled trials (RCTs) with low-quality retrospective studies are in the previous meta-analysis. Moreover, several recent RCTs with adequate power and long-term follow-up have been published and involve new evidence [6, 7].

Therefore, to clarify these ambiguous findings, we performed a meta-analysis, comparing the two techniques for the treatment of degenerative lumbar diseases.

## Materials and methods

### Search strategy

**Fig. 1:**
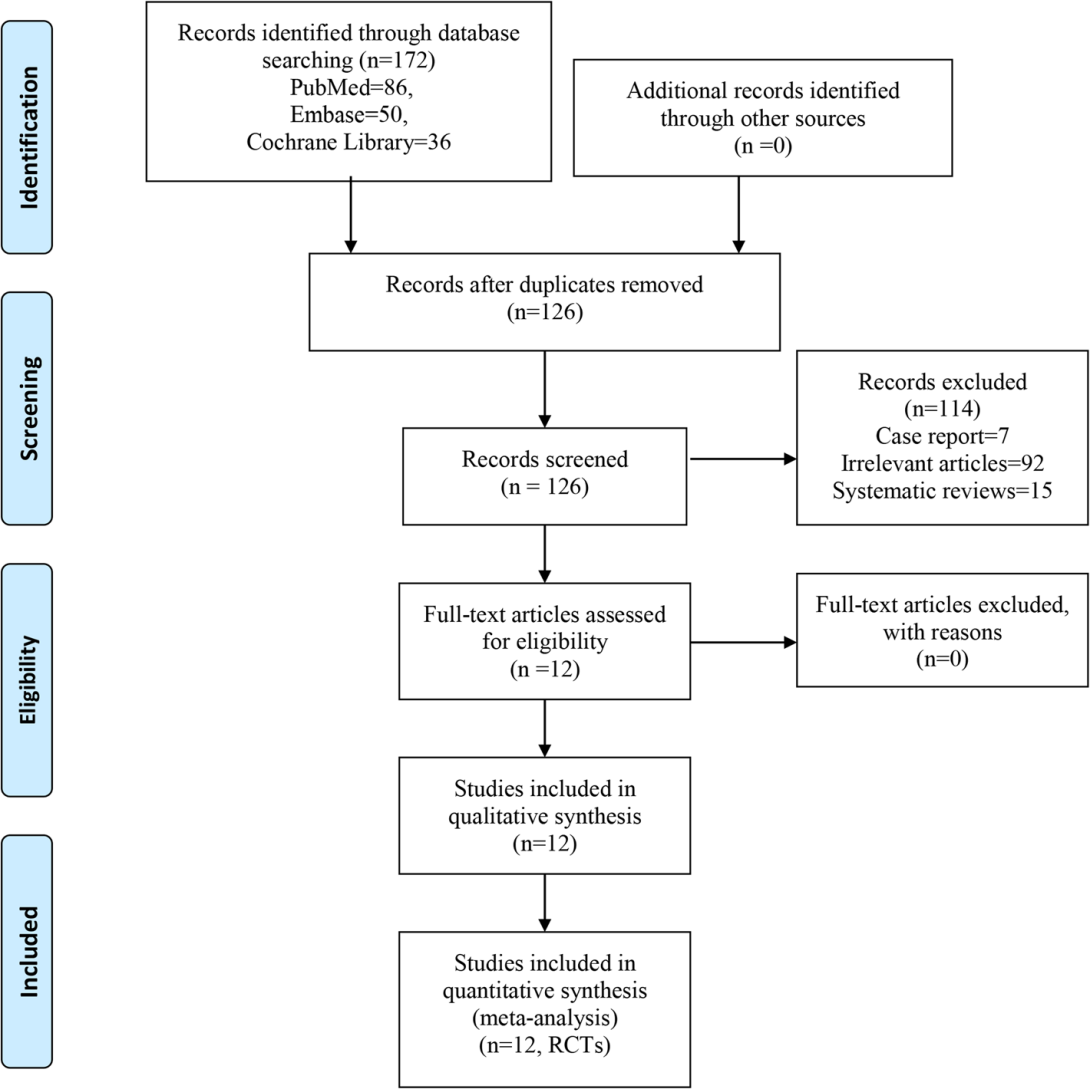
Flow of trials through the meta-analysis

Two reviewers performed an electronic literature search for RCTs or prospective clinical controlled studies comparing the unilateral pedicle screw fixation and bilateral pedicle screw fixation for lumbar degenerative diseases. The electronic databases include PubMed, Embase, and the Cochrane Library up to August 2018. No language or date restrictions were applied. The following terms were used as keywords: ((unilateral pedicle screw fixation) AND bilateral pedicle screw fixation) AND lumbar degenerative diseases. In addition, further articles were obtained by reviewing the references of the selected articles. The detail retrieval process is shown in Fig. 1.

### Inclusion criteria

Randomized controlled trials were included if they met the PICOS criteria as follows: Population: patients with lumbar degenerative diseases; Intervention: unilateral pedicle screw fixation; Comparator: bilateral pedicle screw fixation; Outcomes: fusion rate, visual analog scale (VAS) at final follow-up, Oswestry Disability Index (ODI), Japanese Orthopedic Association scores (JOA), short-form health survey (SF-36), total blood loss, operation time, length of hospital stay, complications, and cage migration; Study design: RCTs or prospective clinical controlled studies. Fusion rate was assessed by the digital upright lateral radiographs according to the Bridwell-Lenke grading [8].

### Data extraction

Two reviewers independently retrieved the relevant data from articles using a standard data extraction form. The extracted data included publication date, authors, country, number of patients, mean age of patients, follow-up duration, type of operation, and surgical segments. For missing data, such as standard deviations, we tried to get it by contacting the original author first. If it did not work, we calculated the missing standard deviations from other available data such as standard errors or the formulas in Cochrane Handbook for Systematic Reviews of Interventions. Two reviewers extracted the data independently, and any disagreement was discussed until a consensus was reached.

### Risk of bias and quality assessment

The methodological bias and quality of included studies were assessed by The Cochrane Collaboration’s tool for assessing the risk of bias according to the Cochrane Handbook for Systematic Reviews of Interventions. It is a two-part tool with seven specific domains: sequence generation, allocation concealment, blinding of participants and personnel, blinding of outcome assessment, incomplete outcome data, selective outcome reporting, and other sources of bias.

### Statistical and subgroup analysis

Stata 12.0 (Stata Corp., College Station, TX) was used to perform the meta-analysis. We used weighted mean difference (WMD) and 95% confidence interval (CI) to assess continuous variable outcomes. For dichotomous outcomes, a risk ratio (RR) with a 95% CI was presented. Heterogeneity between studies was assessed by *I*^2^ and *χ*^2^ test. While *I*^2^ < 50% and *P* > 0.1, we used a fixed effects model to evaluate; otherwise, a random-effects was used. In addition, subgroup analysis (1-segment, 2-segment, 1-segment or 2-segment; TLIF or MIS-TLIF) and sensitivity analysis were performed to explore the source of heterogeneity when heterogeneity existed.

## Results

### Search results

Initially, a total of 172 studies were searched via the databases and other sources (e.g., references). And 46 of the 172 studies were excluded by Endnote Software (Version X7, Thompson Reuters, CA, USA). After reading the full text of the 126 remaining studies in detail, 114 studies were excluded based on the titles and abstracts of these papers. Finally, 12 RCTs [9–20] with 808 patients (unilateral pedicle screw fixation = 393, bilateral pedicle screw fixation = 415) were included in our meta-analysis (published between 2007 and 2014). The general characteristic of the included studies can be seen in Table 1. All of the included studies were published from the year 2007. Seven studies were originated from China, two from the USA, others were from other countries. Sample size ranged from 16 to 56. Mean age ranged from 53.2 to 67 years.

**Table 1:** General characteristic of the included studies

Author	Country	No. of patients (*n*)		Mean age (years)		Follow-up (month)		Type of operation	Surgical segments
		Unilateral	Bilateral	Unilateral	Bilateral	Unilateral	Bilateral		
Aoki [9]	Japan	25	25	66.2	65.6	31	31.2	TLIF, capstone cage, autograft	1-segment
Choi [10]	South Korea	26	27	53.4	56.2	27.5	28.9	TLIF, capstone cage, autograft	1-segment
Duncan [11]	USA	46	56	53.5	55.7	25.1	25.1	TLIF, PEEK cage, autograft/allograft	1-segment
Feng [12]	China	20	20	53.8	53.2	24	24	TLIF, capstone cage, autograft	1-segment
Fernandez-Fairen [13]	Spain	40	42	61.4	60.8	55.6	59.7	Cage, autograft	1-segment, 2-segments
Lin [14]	China	43	42	67	65.5	26	26	MIS-TLIF, capstone cage, autograft	1-segment
Xie [15]	China	56	52	56.2	55	36	36	TLIF, capstone cage, autograft	1-segment, 2-segments
Xue [16]	China	37	43	57.1	58.2	25.3	25.3	TLIF, capstone cage, autograft	1-segment, 2-segments
Zhang [17]	China	33	35	59.4	55.7	12	12	TLIF, capstone cage, autograft	2-segments
Shen [18]	China	31	34	57.3	58.9	26.6	26.6	MIS-TLIF, capstone cage, autograft	1-segment
Dong [19]	China	20	19	54	56.6	24	24	PLIF, capstone cage, autograft	1-segment
Dahdaleh [20]	USA	16	20	62.2	57.3	12.4	12.4	MIS-TLIF, capstone cage, autograft	1-segment

*TLIF* transforaminal lumbar interbody fusion, *MIS-TIF* minimally invasive transforaminal lumbar interbody fusion

### Risk of bias in included studies

**Fig. 2:**
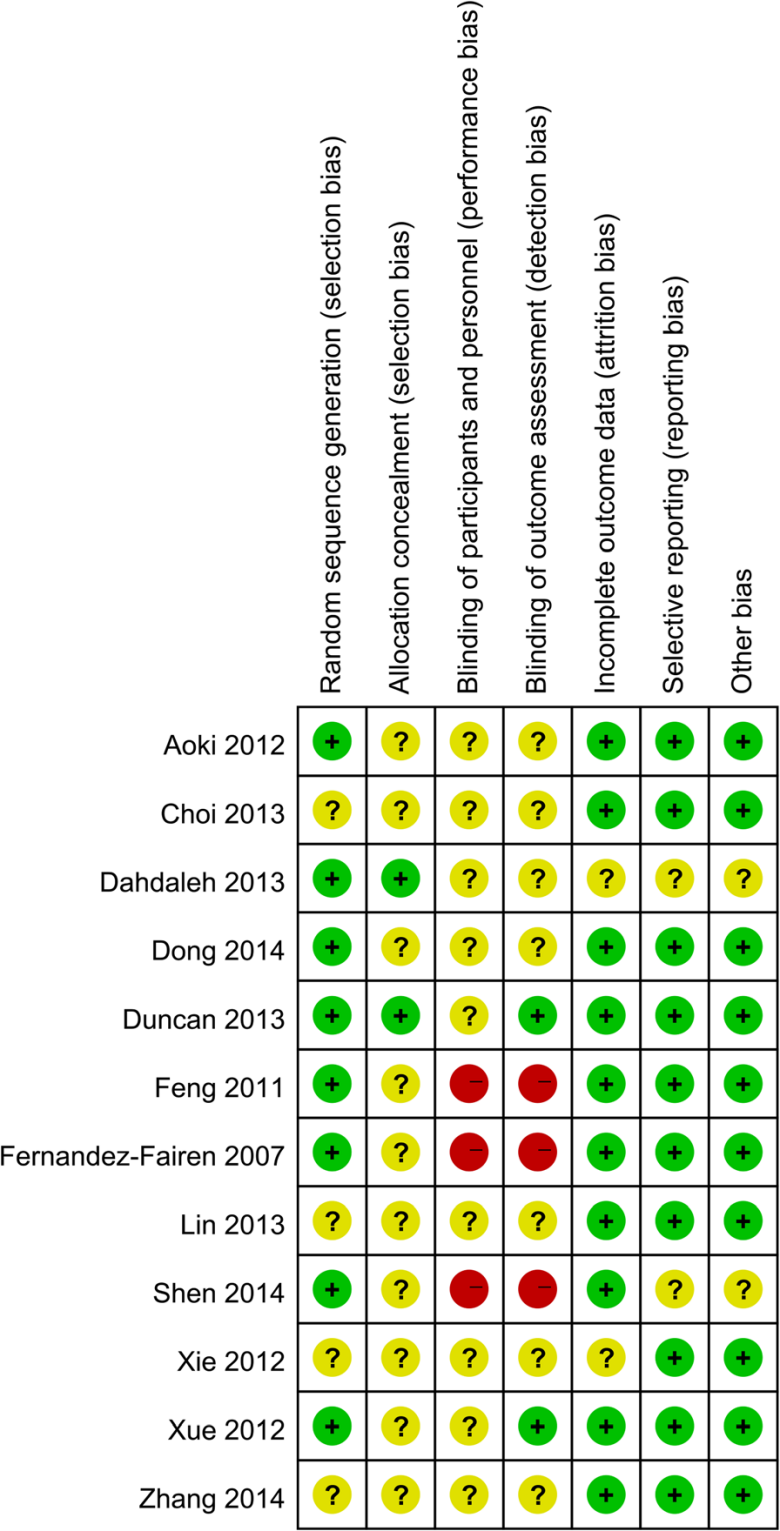
Risk of bias summary of the RCTs

**Fig. 3:**
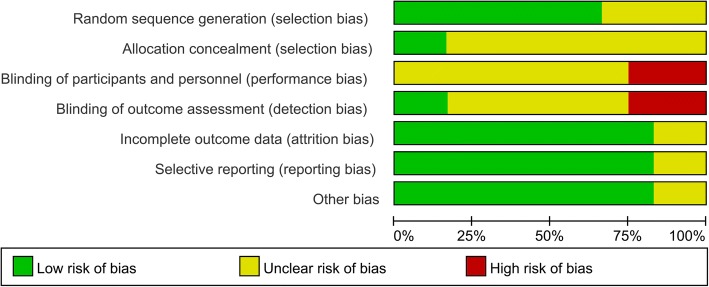
Risk of bias graph of the RCTs

All the included trials were evaluated using the Cochrane Collaboration’s tool for assessing risk of bias in randomized trials and the Grading of Recommendations Assessment, Development and Evaluation Working Group grading scheme. The risk of bias of the RCTs is demonstrated in Figs. 2 and 3 respectively.

### Results of meta-analysis

#### Fusion rate

**Fig. 4:**
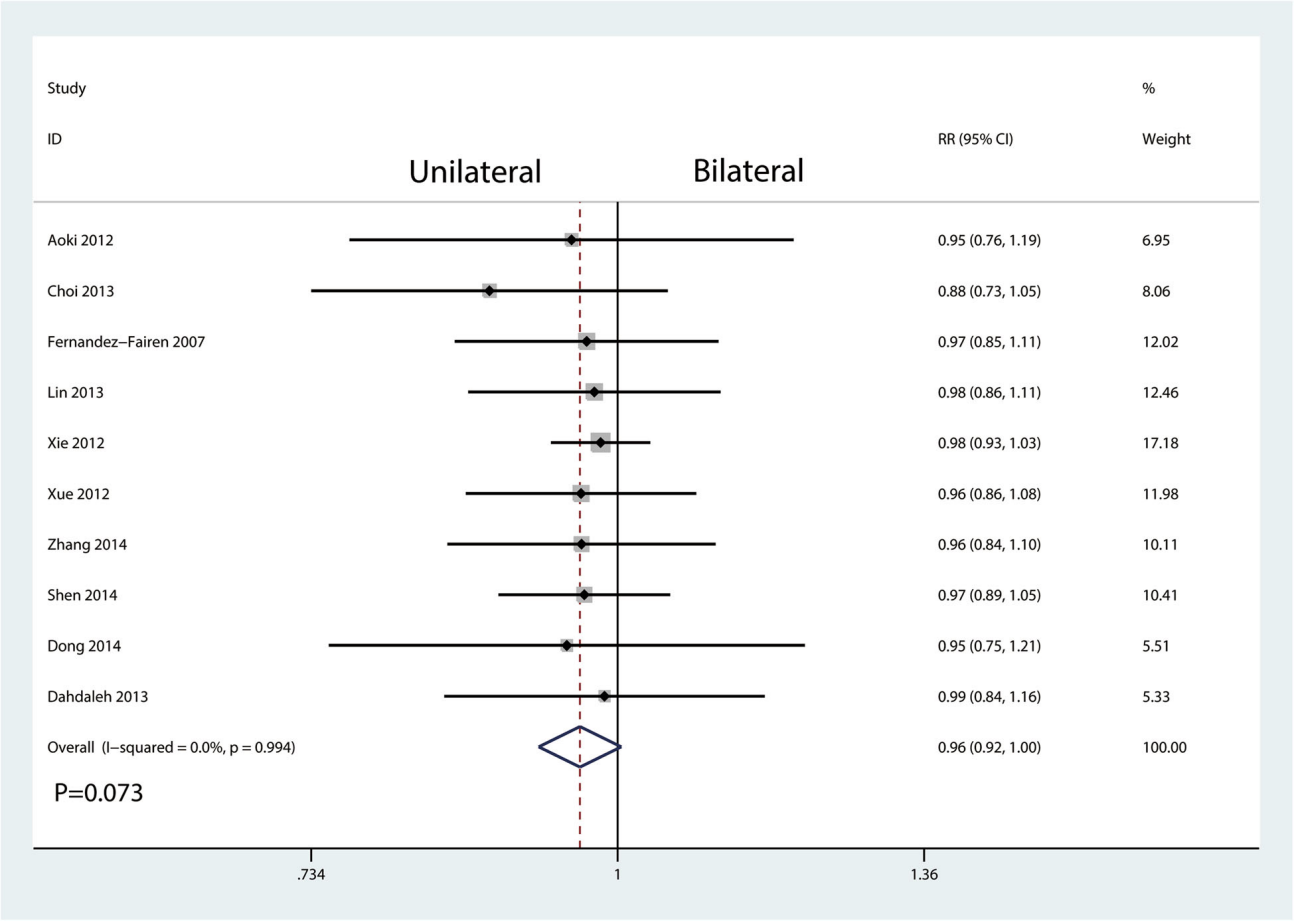
Forest plots of the included studies comparing the fusion rate

Ten studies enrolling 666 patients reported fusion rate postoperatively. No heterogeneity existed between the ten studies (*I*^2^ = 0.0%, *P* = 0.994; Fig. 4). Thus, a fixed effects model was performed. And there was no significant difference between unilateral pedicle screw fixation and bilateral pedicle screw fixation in terms of the fusion rate (RR = 0.96, 95%CI [0.92, 1.00], *P* = 0.073; Fig. 4).

#### VAS at final follow-up

**Fig. 5:**
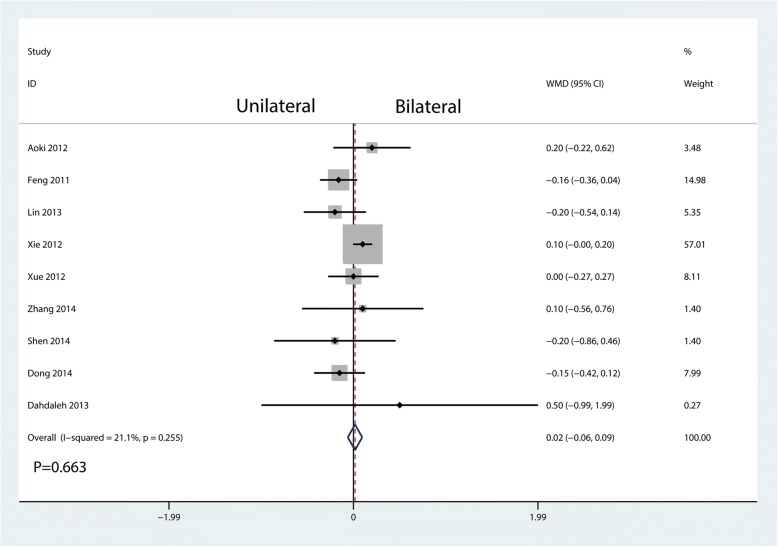
Forest plots of the included studies comparing the VAS at final follow-up

Nine studies enrolling 571 patients reported VAS at final follow-up postoperatively. Slight heterogeneity existed between the nine studies (*I*^2^ = 21.1%, *P* = 0.255; Fig. 5). Thus, a fixed effects model was used. And there was a significant difference between unilateral pedicle screw fixation and bilateral pedicle screw fixation in terms of the VAS at final follow-up (WMD = 0.02, 95%CI [− 0.06, 0.09], *P* = 0.663; Fig. 5).

### ODI, JOA, and SF-36

**Fig. 6:**
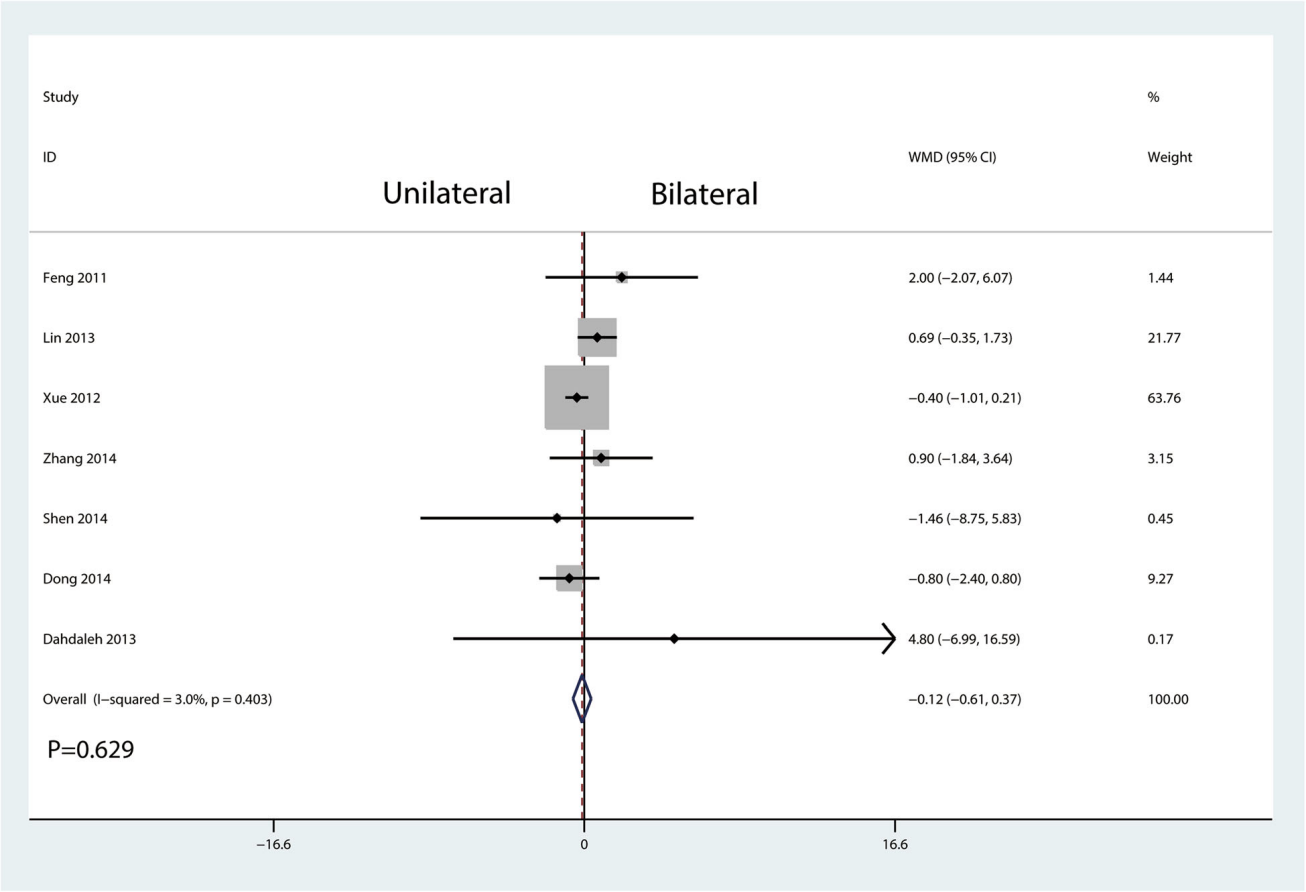
Forest plots of the included studies comparing the ODI

There was no significant difference in the ODI between the unilateral and the bilateral group (WMD = − 0.12, 95% CI = − 0.61 to 0.37; *P* = 0.629, Fig. 6) with little heterogeneity (*I*^2^ = 3.0%, *P* = 0.403; Fig. 6).

**Fig. 7:**
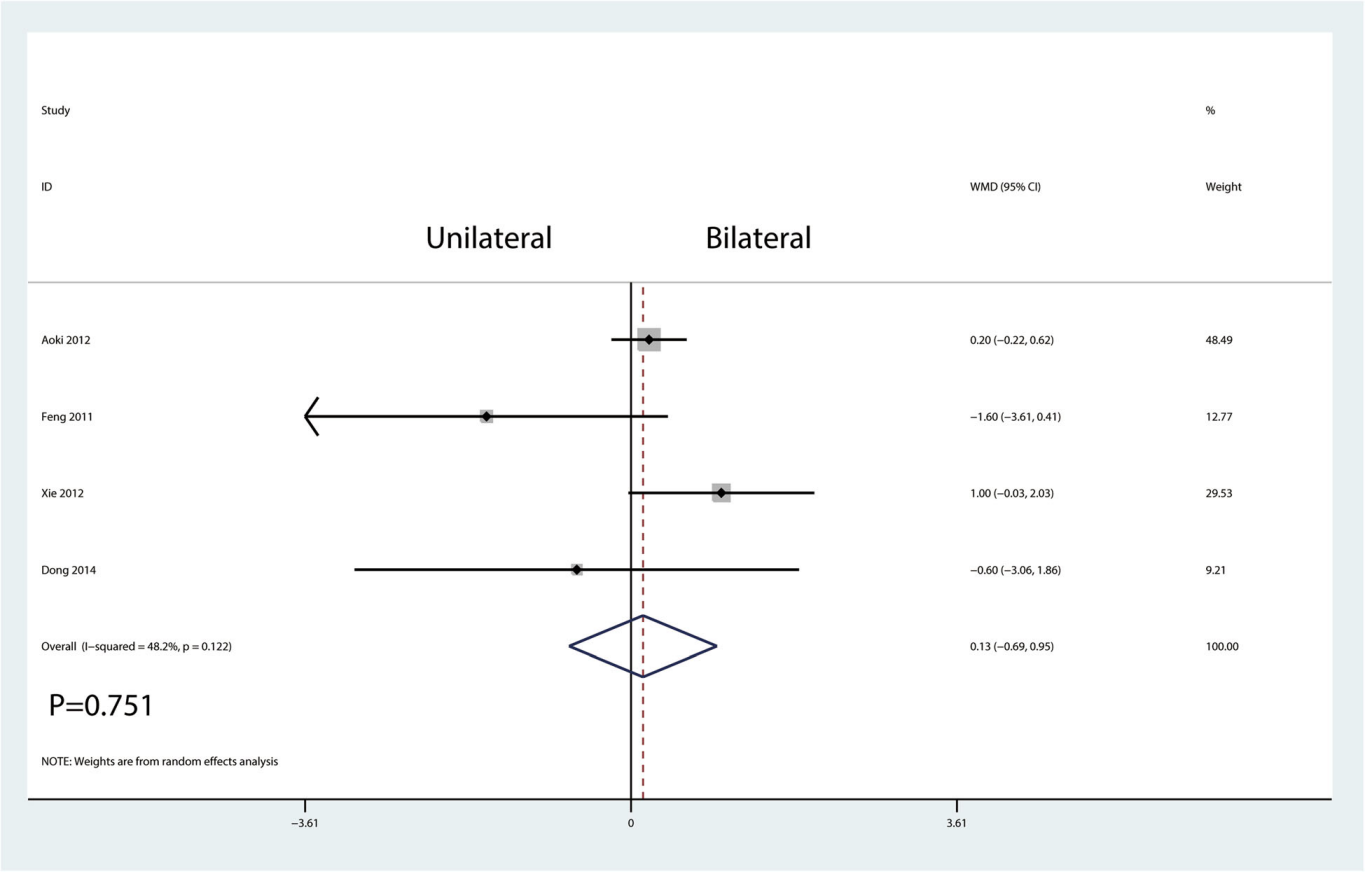
Forest plots of the included studies comparing the JOA

JOA score was reported in four studies, and there was a large heterogeneity between the included studies (*I*^2^ = 48.2%, *P* = 0.122; Fig. 7), the results show that there was no significant difference in the JOA score between the unilateral and bilateral group (WMD =0.13, 95% CI = − 0.69 to 0.95; *P* = 0.751).

**Fig. 8:**
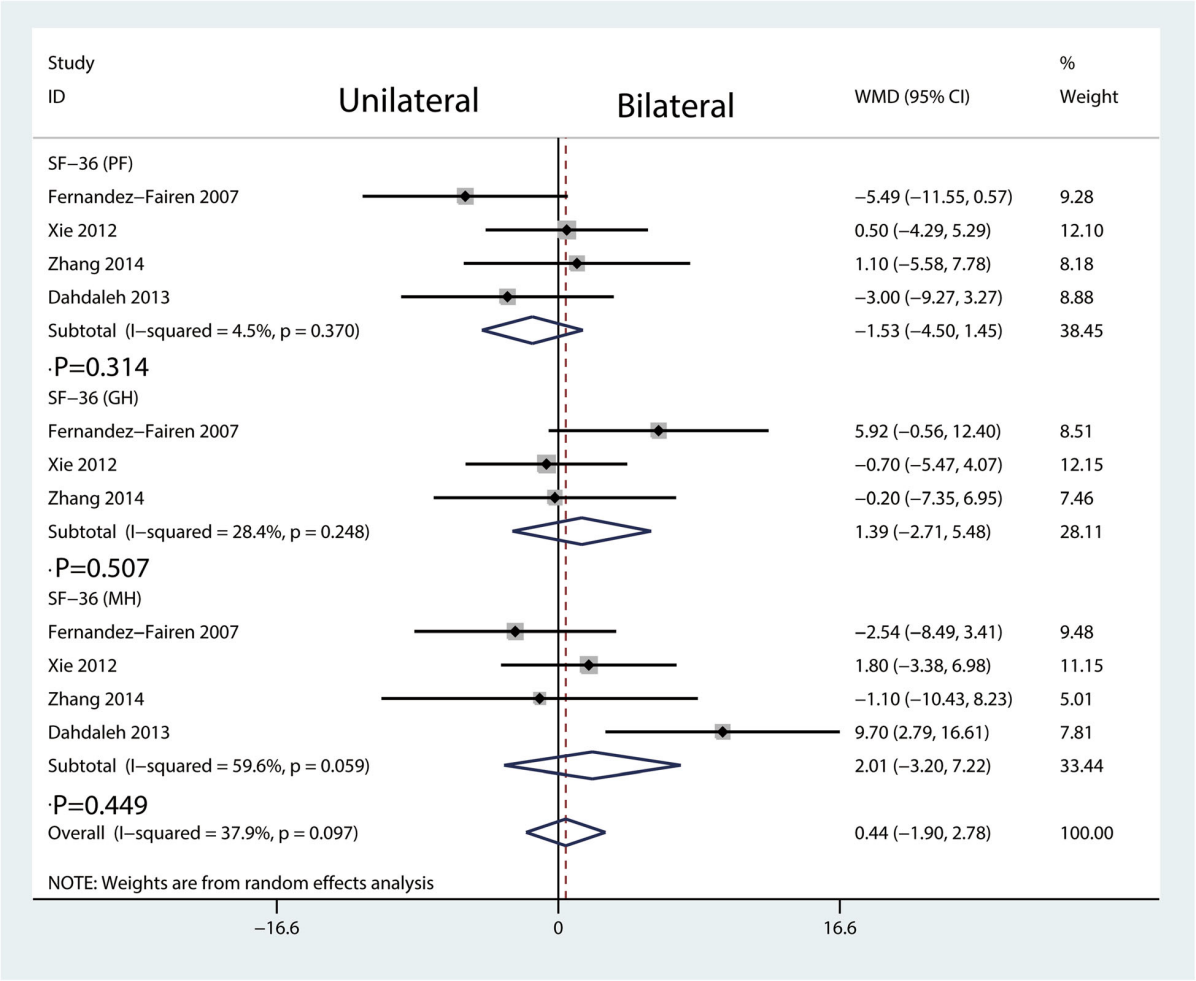
Forest plots of the included studies comparing the SF-36

Four studies included reports of SF-36 (PF, GH, and MH) at final follow-up. There was no difference in SF-36 at final follow-up between the two groups (*P* = 0.314, *P* = 0.507, *P* = 0.449, Fig. 8). There was a large heterogeneity in the SF-36 (MH, *I*^2^ = 59.6%, *P* = 0.059; Fig. 8).

### Total blood loss, operation time, and the length of hospital stay

**Fig. 9:**
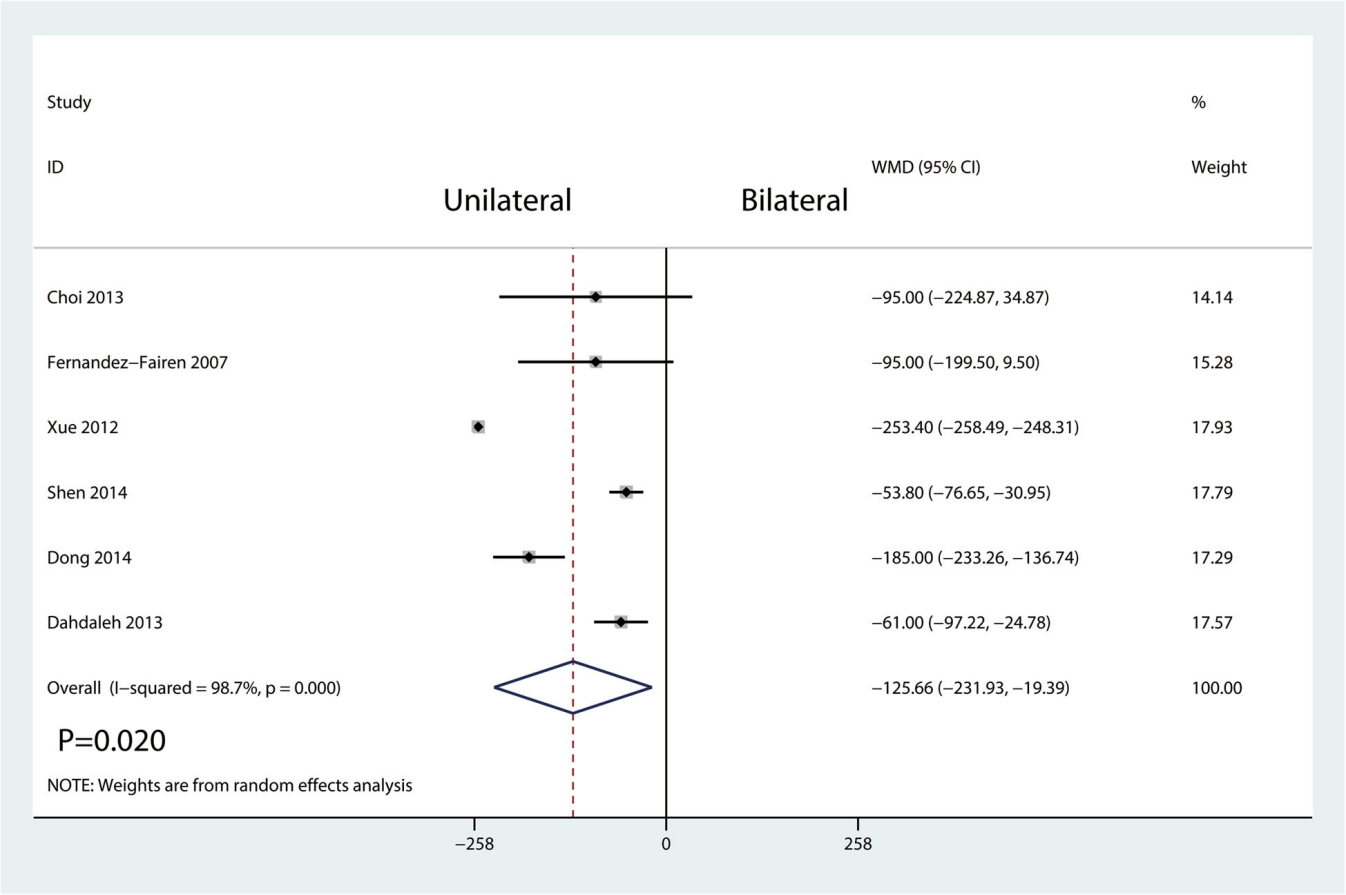
Forest plots of the included studies comparing the total blood loss

Six studies involving 355 patients provided data on total blood loss. Compared with bilateral pedicle screw fixation, unilateral pedicle screw fixation was associated with a reduction of the total blood loss with large heterogeneity (WMD = − 125.66, 95% CI = − 231.93 to − 19.39; *P* = 0.020, *I*^2^ = 98.7%, *P* = 0.000; Fig. 9).

**Fig. 10:**
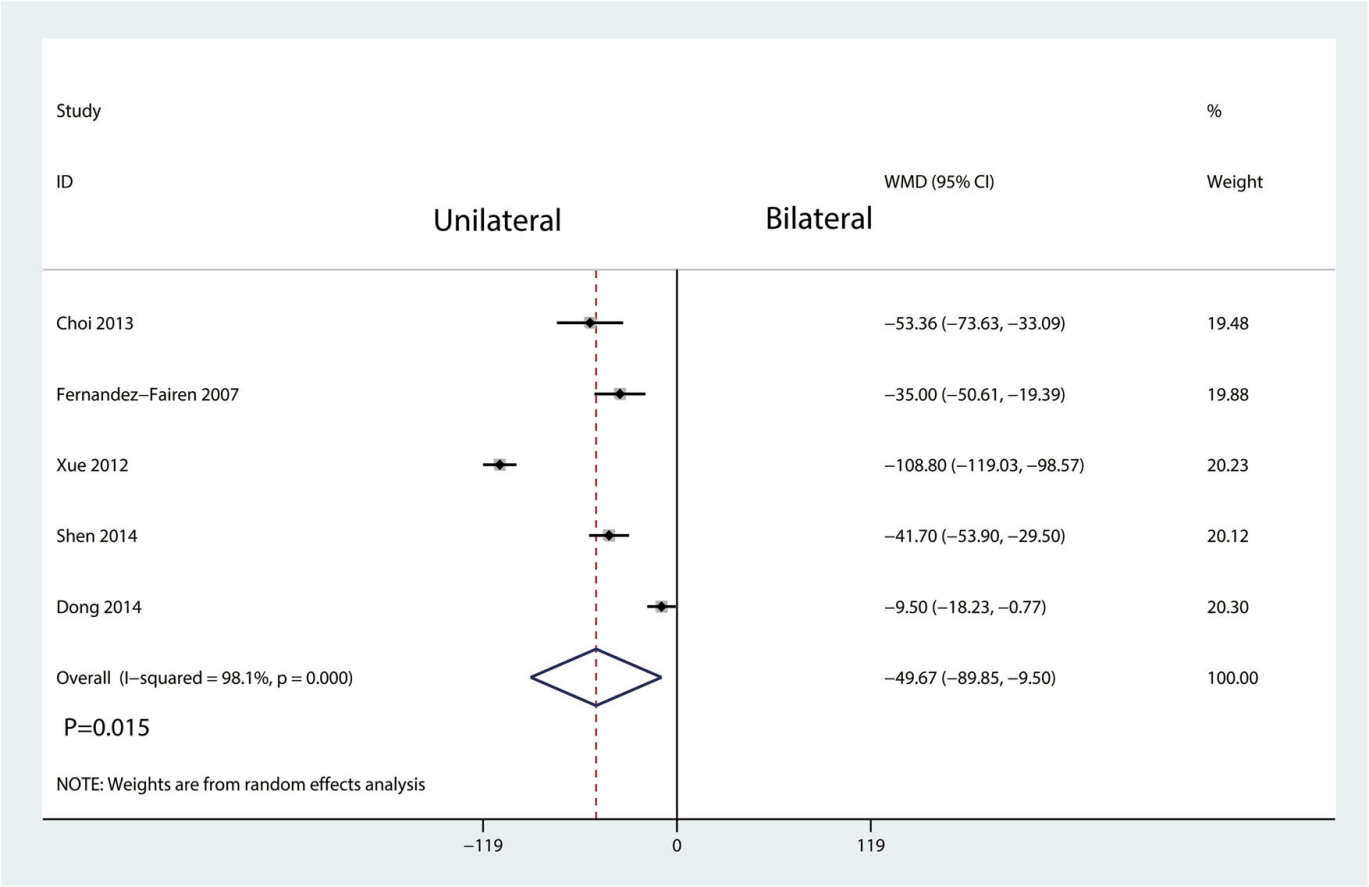
Forest plots of the included studies comparing the operation time

As for the operation time, there was a large heterogeneity between the included five studies (*I*^2^ = 98.1%, *P* = 0.000). Compared with bilateral pedicle screw fixation, unilateral pedicle screw fixation was associated with a reduction of the operation time (WMD = − 49.67, 95% CI = − 89.95 to − 9.50; *P* = 0.015, Fig. 10).

**Fig. 11:**
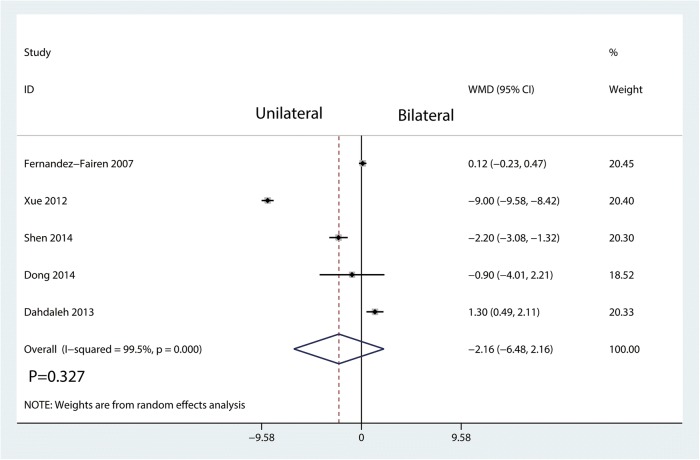
Forest plots of the included studies comparing the length of hospital stay

There was no significant difference between bilateral pedicle screw fixation and unilateral pedicle screw fixation in terms of the length of hospital stay (WMD = − 2.16, 95% CI = − 6.48 to 2.16; *P* = 0.327) with large heterogeneity (*I*^2^ = 99.5%, *P* = 0.000; Fig. 11).

### Complications and cage migration

**Fig. 12:**
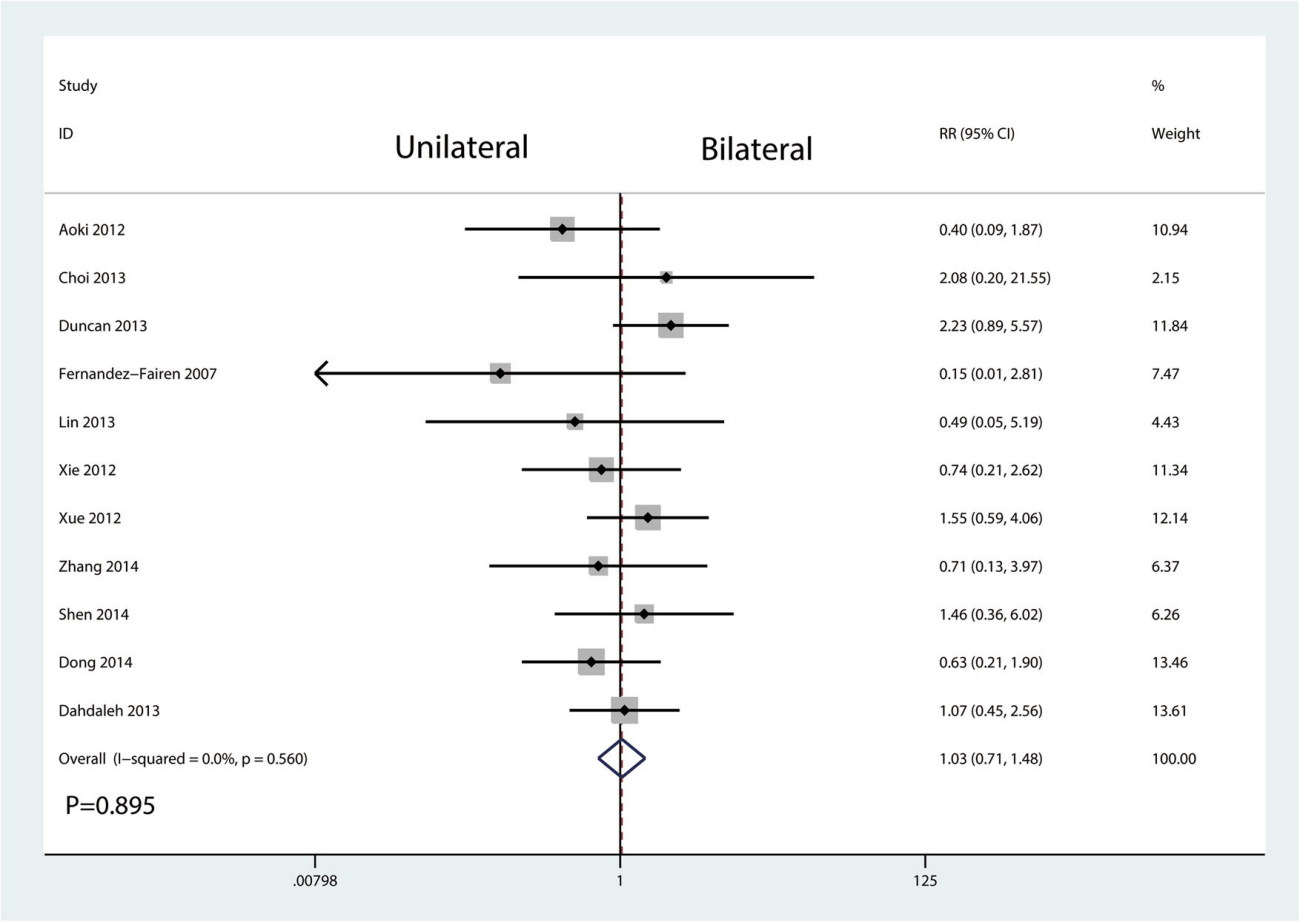
Forest plots of the included studies comparing the complication

Eleven studies involving 768 patients provided data on complications. Compared with bilateral pedicle screw fixation, unilateral pedicle screw fixation has no effects on complications (RR = 1.03, 95% CI = 0.71 to 1.48; *P* = 0.895, Fig. 12).

**Fig. 13:**
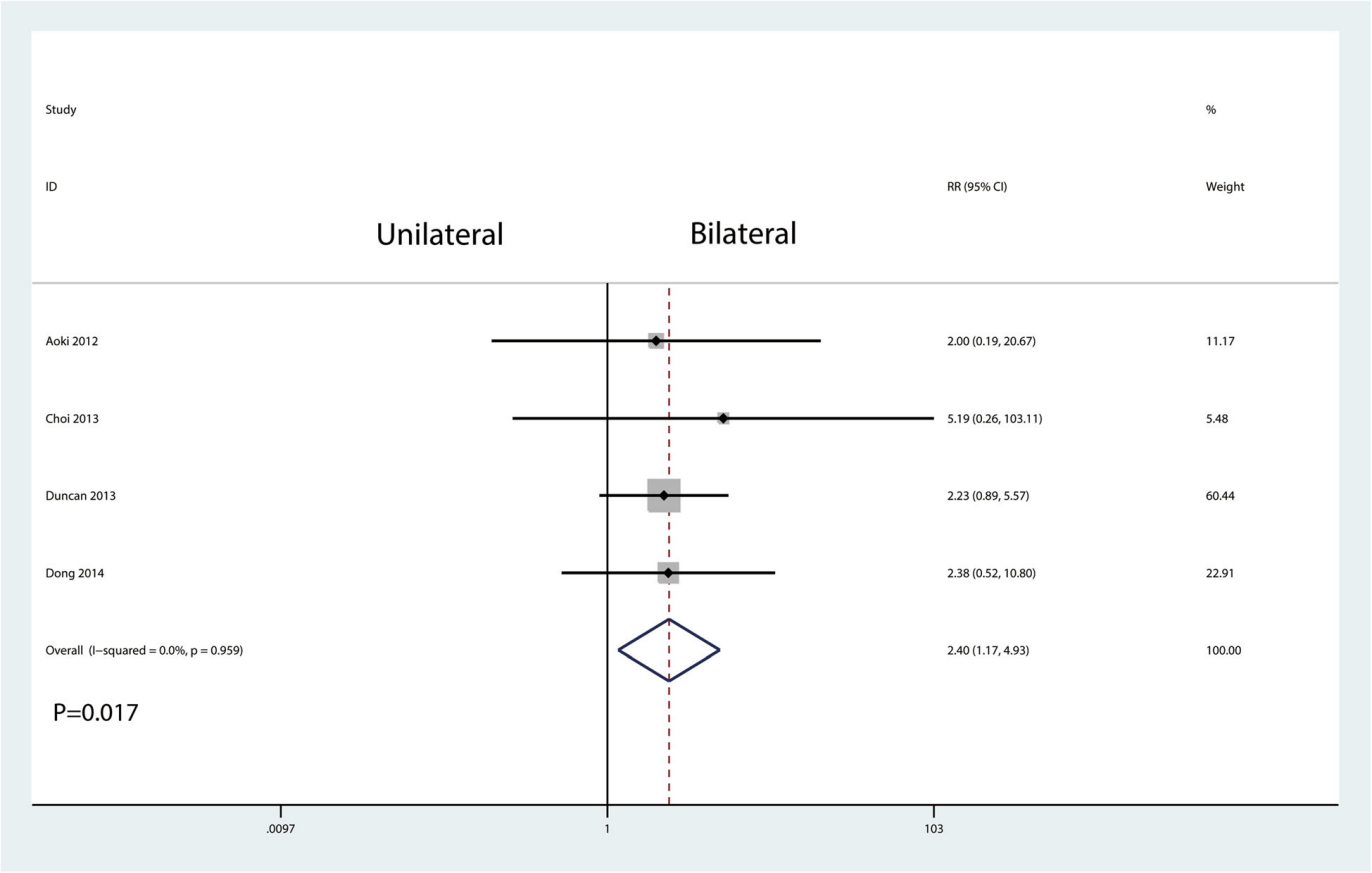
Forest plots of the included studies comparing the cage migration

Four studies involving 244 patients provided data on cage migration. The results showed that unilateral pedicle screw fixation was associated with an increase of the cage migration than bilateral pedicle screw fixation (17.1% vs 7.1%, RR = 2.40, 95% CI = 1.17 to 4.93; *P* = 0.017, Fig. 13).

### Subgroup analysis, sensitivity analysis, and publication bias

**Fig. 14:**
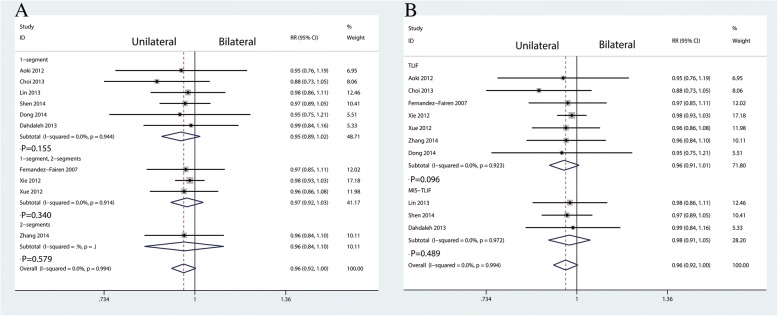
Subgroup analysis for fusion rate according to the surgical segments (**a**) and type of operation (**b**)

**Fig. 15:**
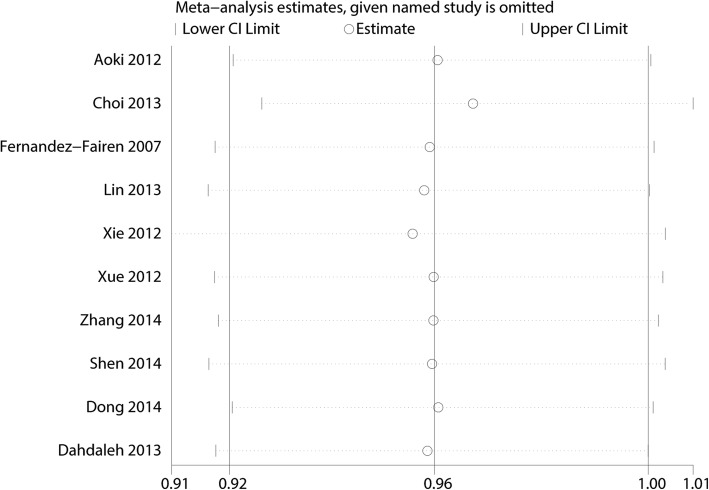
Sensitivity analysis for fusion rate

Figure 14 presents the results of subgroup analyses. The findings of the fusion rate were consistent in all subgroup analyses. We performed a sensitivity analysis to assess the stability of the pooled results. Among most of the studies, the heterogeneity results were not obviously altered after sequentially omitting each study, indicating that our results were statistically reliable (Fig. 15). For the meta-analysis of unilateral pedicle screw fixation versus bilateral pedicle screw fixation on fusion rate, there was no evidence of publication bias by formal statistical tests (Egger test, *P* = 0.56).

## Discussion

### Main finding

Our meta-analysis comprehensively and systematically reviewed the currently available literature and found that (1) unilateral pedicle screw fixation compared with bilateral pedicle screw fixation significantly reduced total blood loss, operation time, and increase the cage migration and (2) unilateral pedicle screw fixation had no benefit on VAS, JOA, ODI, SF-36, and complications compared with bilateral pedicle screw fixation for lumbar degenerative diseases patients.

### Comparison with other meta-analyses

Two relevant meta-analyses about unilateral pedicle screw fixation and bilateral pedicle screw fixation have been published. Although the main finding of our meta-analysis was consistent with most of the outcomes of previous meta-analyses, differences between ours and the previous ones should be noted. First, these previous meta-analyses included no more than six trials and 399 patients. In comparison, our current meta-analysis included 12 RCTs involving 808 patients. With the added statistical power of at least 409 cases, our current meta-analysis was the latest and the most comprehensive one, which generally concurs and further reinforces earlier results of previous meta-analyses. Second, we only included RCTs and thus the selection bias was avoided. Third, we performed subgroup analysis and sensitivity analysis to increase the robustness of our meta-analysis.

We identify the fusion rate as the main outcome. The results showed that unilateral pedicle screw fixation has a similar fusion rate with bilateral pedicle screw fixation at the final follow-up. There is a heated discussion about the biomechanics stability of unilateral pedicle screw fixation. Han et al. [21] conducted a meta-analysis and they found that there was significantly a higher fusion rate in the bilateral group than in the unilateral group. A major concern about this meta-analysis was that they mixed RCTs with non-RCTs. Xiao et al. [6] found that there was no statistically significant difference in terms of the fusion rate. However, only eight RCTs involving 545 patients were included. Chen et al. [22] performed a biomechanical study and found that unilateral fixation with cage implantation was sufficient to maintain the stability of the lumbar spine. The reason may be that unilateral pedicle screw fixation with cage fusion provides enough biomechanical stability for intervertebral fusion. It was probably considered as a two-point fusion in the unilateral screw fixation, while it was a three-point fusion in the bilateral screw fixation. That means that two-point fusion has a similar fusion rate when compared with three-point fusion.

We then compared unilateral and bilateral groups in terms of the clinical and functional outcomes (VAS, JOA, ODI, and SF-36). The ODI was used to assess a patient’s disability during the activities of daily living. The results showed that unilateral pedicle screw fixation has similar outcomes with bilateral pedicle screw fixation. Lin et al. [23] found that when patients in the unilateral pedicle fixation group are compared with the bilateral pedicle screw fixation group on the VAS, ODI scores demonstrated no significant differences. Xiao et al. [6] drawn a similar conclusion with our results. They found that unilateral pedicle screw fixation with cage fusion achieves a similar VAS, ODI, and SF-36 scores with bilateral pedicle screw fixation.

Operation time, blood loss, and the length of hospital stay were took for evaluating surgical trauma and economic costs in this study. The results showed that unilateral pedicle screw fixation was associated with a reduction of the operation time and blood loss than bilateral pedicle screw fixation. No significant difference was found for the length of hospital stay. Since unilateral pedicle screw fixation only needs dissection on one side of the soft tissue and paravertebral muscles and insertion on the side of the pedicle screw, it can accordingly reduce the operation time and blood loss as compared with bilateral pedicle screw fixation [24]. Moreover, lesser soft tissue dissection may allow for early functional recovery. In theory, unilateral pedicle screw fixation may be related with the short length of hospital stay. In the current meta-analysis, we found no significant difference between the two groups in terms of the length of hospital stay.

There was no significant difference between the occurrence of complication. There was no significant heterogeneity between the included studies. Eliades et al. [5] found that bilateral pedicle screw fixation increases the stability of the spine, and it also increases the implant costs and the incidence of neurologic complications. And, unilateral pedicle screw fixation was associated with an increase of the cage migration than bilateral pedicle screw fixation. Many studies have reported that unilateral fixation is not stable enough to prevent fusion cage migration [11]. The reason may be that unilateral screw fixation inherently results in asymmetry. Therefore, the cage should be inserted obliquely into the disk space, and the cage of the anterior part should cross the midline of the vertebral body to support the contralateral anterior column.

The current study had several limitations. (1) Patients with different phases of follow-up were included. There was marked heterogeneity among the included studies in terms of total blood loss, length of hospital stay, and JOA. (2) The type of operation (including TLIF and MID-TLIF) and surgical segments varied between studies. (3) Blinding of the participant was unclear and this bias could not be avoided. (4) Some data for comparisons were not originally available but were calculated by estimation, leading to other bias.

## Conclusion

Unilateral pedicle screw fixation and bilateral pedicle screw fixation have similar fusion rates when treating for lumbar degenerative diseases. However, compared with the bilateral pedicle screw fixation, the unilateral pedicle screw fixation significantly reduced total blood loss and operation time for lumbar degenerative diseases. The use of unilateral pedicle screw for lumbar degenerative diseases increases the cage migration. There were no significant differences between the unilateral pedicle screw fixation and bilateral pedicle screw fixation in terms of the VAS at final follow-up, JOA, ODI, and SF-36. Unilateral pedicle screw fixation is recommended as the optimal surgical method for lumbar degenerative diseases.

## Abbreviations

CI: Confidence interval
JOA: Japanese Orthopedic Association scores
ODI: Oswestry Disability Index
RCTs: Randomized controlled trials
RR: Risk ratio
SF-36: Short-form health survey
VAS: Visual analog scale
WMD: Weighted mean difference
